# Intramuscular Neural Arborization of the Latissimus Dorsi Muscle: Application of Botulinum Neurotoxin Injection in Flap Reconstruction

**DOI:** 10.3390/toxins14020107

**Published:** 2022-01-30

**Authors:** Kyu-Ho Yi, Hyung-Jin Lee, Kyle K. Seo, Hee-Jin Kim

**Affiliations:** 1 COVID-19 Division, Wonju Public Health Center, Wonju 26417, Korea; kyuho90@korea.kr; 2 Division in Anatomy and Developmental Biology, Department of Oral Biology, Human Identification Research Institute, BK21 PLUS Project, Yonsei University College of Dentistry, 50-1 Yonsei-ro, Seodaemun-gu, Seoul 03722, Korea; leehj221@yuhs.ac; 3 Modelo Clinic, Seoul 06011, Korea; doctorseo@hotmail.com; 4 Department of Materials Science & Engineering, College of Engineering, Yonsei University, Seoul 03722, Korea

**Keywords:** myocutaneous flap, latissimus dorsi abdominis muscle, botulinum neurotoxin, Sihler’s method

## Abstract

Postoperative pain after breast reconstruction surgery with the latissimus dorsi flap is a common occurrence. Botulinum neurotoxin (BoNT) injection during surgery is effective in reducing postoperative pain. This study aimed to determine the most appropriate locations for BoNT injection. A modified Sihler’s method was performed on the latissimus dorsi muscles in 16 specimens. Intramuscular nerve arborization was noted under the landmark of the medial side surgical neck of the humerus to the line crossing the spinous process of T5 and the middle of the iliac crest. The latissimus dorsi muscles were divided into medial, middle, and lateral segments with 10 transverse divisions to give 10 sections (each 10%). Intramuscular nerve arborization of the latissimus dorsi muscle was the largest from the medial and lateral part of the muscle ranging from 40 to 60%, middle part from 30 to 60% and medial, middle and lateral part from 70 to 90%. The nerve entry points were at the medial and lateral part with 20–40% regarding the medial side of surgical neck of the humerus to the line crossing spinous process of T5 to the middle of iliac crest. These outcomes propose that an injection of BoNT into the latissimus dorsi muscles should be administered into specific zones.

## 1. Introduction

Breast cancer is one of the most common cancers, accounting for over 30% of all cancers in women. The prevalence of breast cancer continues to increase annually, but mortality has declined to 15% because of early detection as well as advanced medical treatments [1]. After surgical operations, reconstruction with breast augmentation allows satisfaction of the patient’s aesthetic desires. Annually, the total number of mastectomy patients who opt to undergo post-mastectomy breast reconstruction continues to grow [2].

Breast reconstruction using latissimus dorsi (LD) flap is a commonly used autologous implant substance [3,4,5,6,7,8,9]. The benefits of using autologous tissue involve exceptional aesthetic results and for the prevention of difficulties associated with foreign substances [10]. Post-flap surgery is supposedly somewhat painful for the patients, which requires extremely supervised pain management [11]. The leading cause of the pain in the flap is muscular contraction and spasms after the transplantation surgery [12,13,14]. Prior studies have reported that BoNT injections after harvesting the flap by free hand or neurolysis in patients with myocutaneous flap reconstruction surgery, significantly eases muscular contraction and reduces the patient’s pain [7,15,16,17,18]. Furthermore, studies have shown that a BoNT in the myocutaneous flap had a positive impact by reducing necrosis percentages, enhancing arterial cross-sectional diameters, as well as the microvascular densities [19]. Additionally, researches have stated definite consequences of BoNT on flap survivability and tissue blood flow [20,21,22].

The BoNT mechanism of action is the permanent blockage of acetylcholine release for up to 12–24 weeks on the motor end plate. Increasing flap survival rate is the main purpose in transplantation surgery, thereby, the research of novel approaches to reduce the flap necrosis rates is a meaningful issue. The positive effects of BoNT for the survival of the muscle and fatty tissues of the flaps and the mechanism of action were previously assessed [20,23,24].

Up to the present, BoNT injections are considered among the most efficient and reliable therapeutic alternatives for alleviating muscular contraction [23,24,25]. The action of the BoNT depends on its uptake into the presynaptic membrane of the motor neuron at the motor end plate. Consequently, the injection should be administered into the densely located motor-end-plate regions [26,27,28]. BoNT therapies targeting the neural arborized zone, the area with the most motor-end-plates, are clinically examined to verify their effectiveness on psoas major muscles and biceps brachii [29,30]. It was established in these trials that locations with densely located motor-end-plate administered injections resulted in much greater muscle reduction in volume than conventional injection treatments [29,30].

Practitioners have to be aware that high-level doses of BoNT can cause neurotoxin to disperse to adjunct muscles, which will have unfavorable paralysis [31,32]. In addition, high-level doses and recurrent injections of BoNT produce the antibodies that decrease effectiveness [31,32,33]. The development of neutralizing antibodies in repetitive BoNT is correlated with injection intervals, cumulative dose and depending on the toxin products [34]. Thus, to lessen the undesirable effects and enhance efficiency, LD muscle BoNT injection should be targeted into the neural arborized zones. Studies have focused primarily on identifying the intramuscular arborized zones and guidance injectable points for BoNT treatments [35,36,37,38,39,40,41,42,43,44,45].

Up to this point, no articles regarding the LD muscles have clarified the intramuscular neural arborization. Dissection analyses is challenging due to the obstacle of naked-eye tracking of the microscopic distribution of intramuscular nerve, and the probability of damage to the nerve [46,47]. In the present study, Sihler staining method, a whole-mount staining method that efficiently uncovers distributions of the intramuscular nerve devoid of damaging the nerves, was utilized.

This study aimed to clarify the intramuscular nerve arborizations of the LD muscles by applying the Sihler staining method. Findings of this study will propose secure and effective BoNT injection points on the LD flaps.

## 2. Results

### 2.1. Intramuscular Arborization Patterns of the LD Muscle

**Figure 1:**
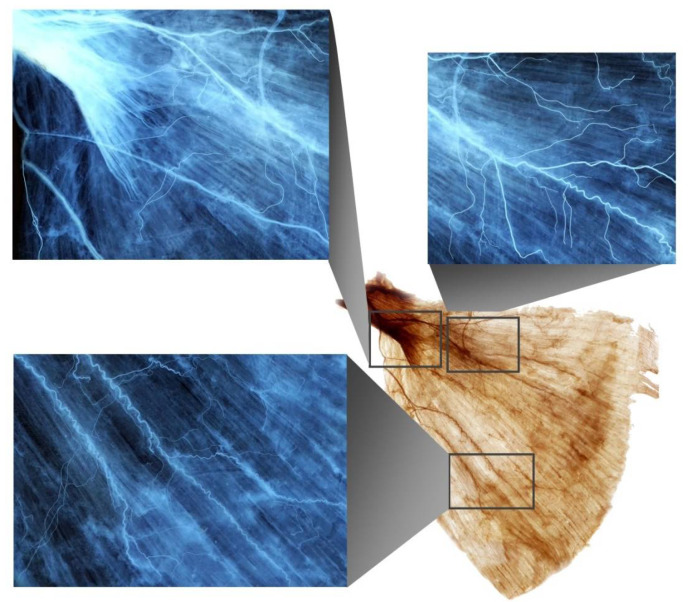
A Sihler’s stained latissimus dorsi muscle with enlarged panels showing intramuscular arborizations.

Fourteen out of 16 LD muscles exhibited intramuscular nerve arborization most extensive at the medial and lateral sections with 40–60%, middle region with 30–60% and medial, middle and lateral parts with 70–90%, regarding the medial side of the surgical neck of the humerus to the line crossing the spinous process of T5 to the middle of the iliac crest (Figure 1). One had the largest at medial 40–60%, lateral part with 50–60%, middle part with 30–60% and medial, middle and lateral parts with 70–90%. Another had the largest arborization at medial 50–60%, lateral part with 50–60%, middle part with 30–60% and medial, middle and lateral parts with 70–90%.

### 2.2. Nerve Entry Point of the LD Muscle

The thoracodorsal nerve divided into 3–4 extramuscular branches before entering the muscle. Fourteen of the 16 LD muscles had the nerve entry point at the medial and lateral part with 20–40%, regarding the medial side of the surgical neck of the humerus and the line crossing the spinous process of T5 to the middle of the iliac crest. The other two had the nerve entry point at the middle and lateral part with 30–40%.

## 3. Discussion

The LD muscle is a superficial muscle which originates from the T5 to L5 spinous processes of the vertebrae, thoracolumbar fascia, 9th to 12th rib, iliac crest and inserts into the intertubercular groove of the humerus. The muscle acts as the shoulder extensor, adductor and internal rotator. The nerve supply is via the thoracodorsal nerve running deep to the muscle [48,49].

The LD flap is one of the most widely used flaps in reconstructive surgery, owing to its wide vascular diameter and lengthy dependable size [9,50]. Common complications after LD flap breast reconstruction surgery may involve muscular contraction causing postoperative pain and breast contour deformities [48,51]. Several studies have suggested that intraoperative resection of the thoracodorsal nerve should be performed to prevent involuntary LD muscle spasm following breast reconstruction. However, this denervation procedure is not always effective and there is the possibility of flap pedicle damage and muscular degeneration leading to poor outcomes [48,51]. It is proposed that the intact thoracodorsal nerve can help to maintain maximum flap quality postoperatively over the long term [52]. For alternative methods, BoNT injection has been utilized to prevent breast deformity and postoperative spasmodic pain following LD flap implantation to the subpectoral area [7].

Figus et al. reported that 71 patients with breast reconstruction surgery with LD flap implants classified muscular complications as considerably discomforting, and requested pain management. Postoperative BoNT infiltrations in the LD flap would be effective for resolving or significantly reducing the post treatment discomfort [7]. Trignano et al. [15] reported that in 83 patients with myocutaneous flap reconstruction, BoNT injections significantly reduced the muscular contraction and decreased pain. Schwartz et al. [53] reported that a patient who developed isolated contraction of the LD flap after breast reconstruction was administered a BoNT injection (300 units) into the LD flap under electromyographic (EMG) guidance. Two weeks following the treatment, muscle twitching had ceased.

Several studies revealed that BoNT infiltration of the flap muscle in breast reconstruction surgery had produced prolonged inhibition of muscle contraction and postoperative pain [2,50,51]. Recently, the hypoxia caused by contraction of the flap muscle was shown to be associated with the etiology of the pain [54]. Schweizer et al. demonstrated that BoNT injection increased flap survival through enhanced blood flow and oxygen supply to the tissues [55,56].

The functional aspect of the shoulder joint and impaired shoulder function with spastic paresis following stroke has application for BoNT treatment in LD muscle. Previous studies observed that shoulder internal rotation and adduction from post-stroke spasticity is significantly reduced after application of BoNT in the LD muscles, which enhances the range of shoulder motion, thus improving functional benefit [57].

BoNT interrupts neurotransmission precisely at the motor-end-plates by absorption at the presynaptic membrane [58]. Clinicians therefore need to precisely manage the toxin in the LD muscle and should inject the toxin in close proximity to the site of action, specifically into the intramuscular neural arborized zone of the LD muscle. To date, no study has investigated the intramuscular neural distribution of the LD muscle to ascertain the best sites for administration of BoNT.

If not injected into the specific area it is suspected that higher doses are needed to block the motor endplates. If not injected into the specific zone, high doses of BoNT would be required to be effective, and when high doses of BoNT are injected, accelerated antibody production occurs. In large muscles, such as the LD muscle, multiple and high-dose injections are necessary, and antibody production reduces the subsequent efficacy of the treatment. Furthermore, BoNT readily diffuses through the muscle fascia even at subclinical doses, spreading to nearby muscles and causing unwanted paralysis [59]. These challenges necessitate the administration of BoNT precisely into the neural arborized zone of the LD muscle at lower doses and with fewer injections.

**Figure 2:**
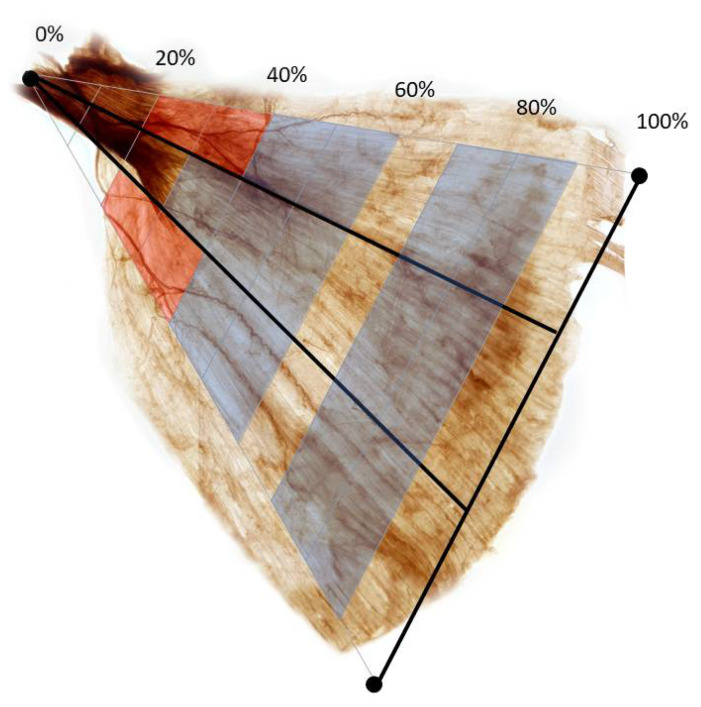
Intramuscular nerve arborization of the latissimus dorsi muscle was the largest from medial and lateral part with 40–60%, middle part with 30–60% and medial, middle and lateral part with 70–90% (blue shaded) and nerve entry point were at the medial and lateral part with 20–40% (red shaded) regarding the medial side of the surgical neck of the humerus to the line crossing spinous process of T5 to the middle of iliac crest.

We propose BoNT injections in the largest nerve arborization at the medial and lateral part with 40–60% and middle part with 30–60% for the LD muscle (Figure 2).

## 4. Materials and Methods

This study was conducted in compliance with the principles set forth in the Declaration of Helsinki. Permission and approval were received from the families of the cadavers before initiating the dissections. All cadavers used in this study were legally donated to the Surgical Anatomy Education Center, Yonsei University College of Medicine (approval code 20-008; approval date: 5 May 2020). A total of 16 LD muscles from Korean cadavers (five men and five women with a mean age of 74.2 years; range, 63–84 years) were dissected at Yonsei University Medical Center from March to May 2020. They were subjected to modified Sihler staining to reveal the intramuscular nerve arborization zones.

**Figure 3:**
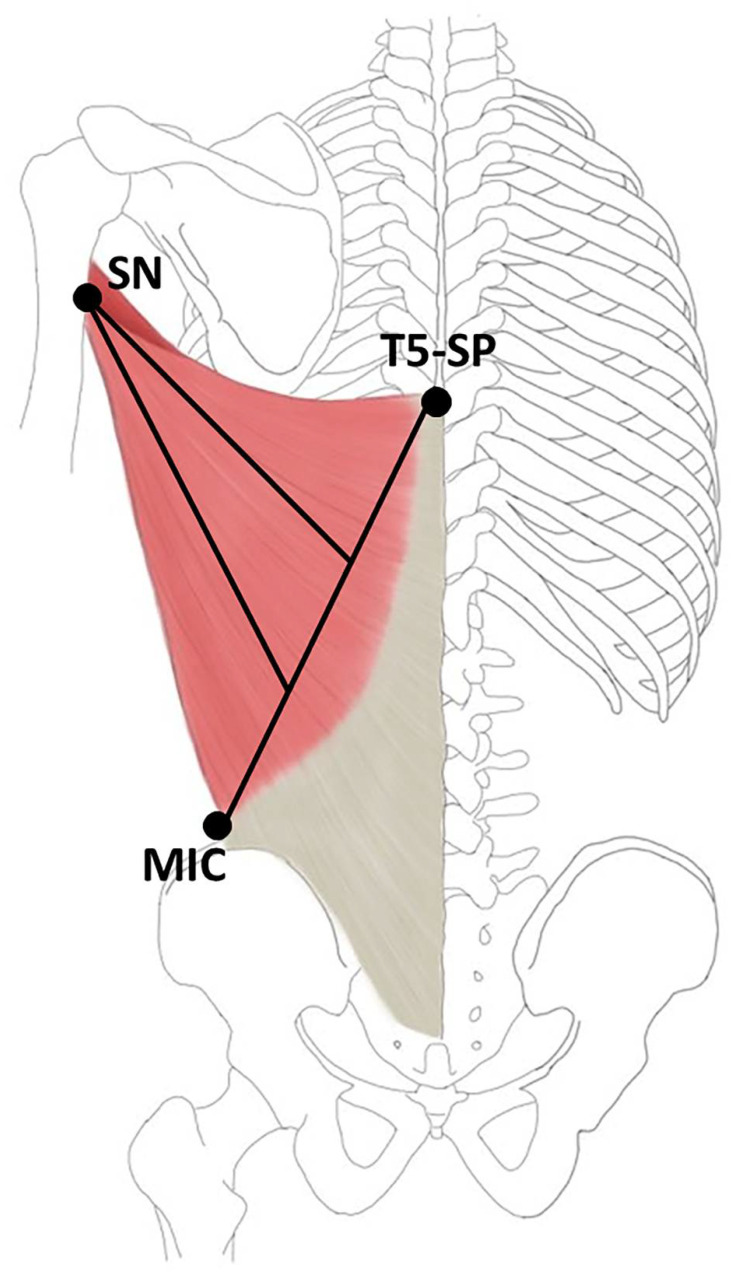
Specimens were harvested from the medial side of the surgical neck of the humerus (SN) to the line crossing spinous process of T5 (T5-SP) to the middle of the iliac crest (MIC).

The LD muscles were exposed to Sihler staining, as adjusted by Liem and Douwe [60]. This method includes multiple steps to obtain a visual representation of the intramuscular neural arborization. After the Sihler stained LD specimens were obtained, they were analyzed by dividing into 30 sections. The LD muscles were divided into 10% from the line crossing the spinous process of T5 to the middle of the iliac crest (0%) to the medial side of the surgical neck of the humerus (100%) and the medial, middle and lateral muscle belly—equal in width (Figure 3).

### Modified Sihler Staining

**Figure 4:**
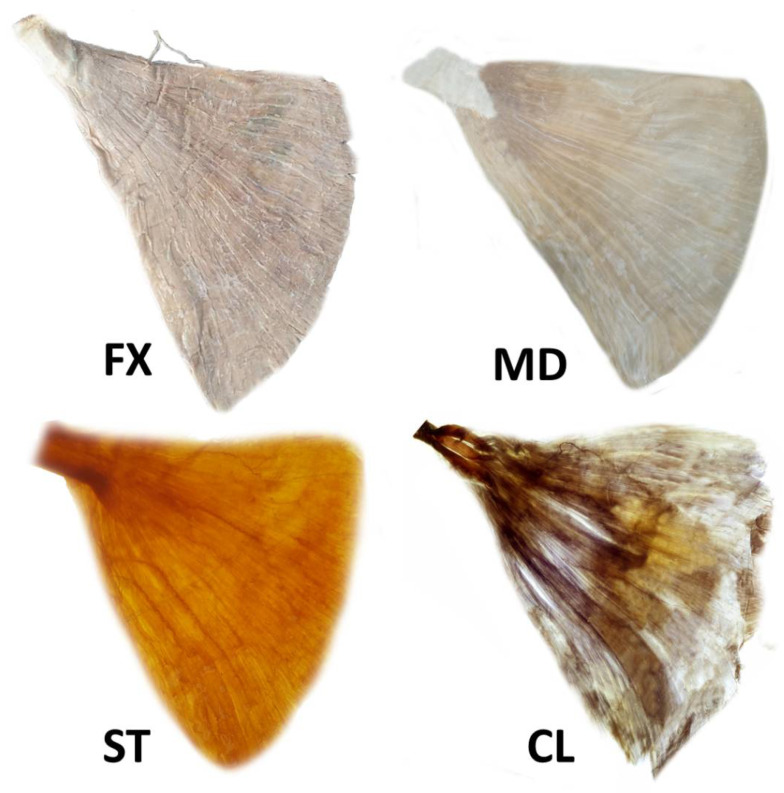
The latissimus dorsi muscle underwent modified Sihler’s method. The method consists of stages of fixation (FX), maceration and depigmentation (MD), decalcification, staining (ST) and clearing (CL).

The steps involved in the preparation of specimens are shown in Figure 4.

Fixation: The harvested LD muscles were fixated in 10% un-neutralized formalin solution for one month. The formalin solution was changed each time the solution became opaque.

Maceration and depigmentation: The fixated LD specimens were washed in flowing water for an hour. Then, the macerated LD specimens were depigmented for 28 days in hydrogen peroxide solution mixed with 3% aqueous potassium hydroxide.

Decalcification: The macerated LD specimens were placed in a Sihler I solution (glycerin, aqueous chloral hydrate and glacial acetic acid) for 24 h.

Staining: Following decalcification, the LD specimens were placed in Sihler II solution (glycerin, aqueous chloral hydrate and acetic acid) for 28 days to stain all the tissue including nerve and muscle fibers.

Destaining: The stained LD specimens were again placed in Sihler I solution for two hours to destain the muscle fibers and leave the stained nerves.

Neutralization: The destained LD specimens were washed in flowing water for 45 min. Subsequently, they were immersed in 0.05% lithium carbonate for 45 min.

Clearing: The neutralized LD specimens were consecutively cleaned with increasing concentrations (20–100%) of glycerin for 5 days.

## Data Availability

Not applicable.
